# The generation of glioma organoids and the comparison of two culture methods

**DOI:** 10.1002/cam4.7081

**Published:** 2024-03-08

**Authors:** Yang Zhang, Yunxiang Shao, Yanyan Li, Xuetao Li, Xuewen Zhang, Qinzhi E, Weichao Wang, Zuoyu Jiang, Wenjuan Gan, Yulun Huang

**Affiliations:** ^1^ Department of Neurosurgery The Fourth Affiliated Hospital of Soochow University Suzhou China; ^2^ Department of Neurosurgery The First Affiliated Hospital of Soochow University Suzhou China

**Keywords:** glioma, organoid, patient‐derived orthotopic xenograft model, stem cell

## Abstract

**Background:**

The intra‐ and inter‐tumoral heterogeneity of gliomas and the complex tumor microenvironment make accurate treatment of gliomas challenging. At present, research on gliomas mainly relies on cell lines, stem cell tumor spheres, and xenotransplantation models. The similarity between traditional tumor models and patients with glioma is very low.

**Aims:**

In this study, we aimed to address the limitations of traditional tumor models by generating patient‐derived glioma organoids using two methods that summarized the cell diversity, histological features, gene expression, and mutant profiles of their respective parent tumors and assess the feasibility of organoids for personalized treatment.

**Materials and Methods:**

We compared the organoids generated using two methods through growth analysis, immunohistological analysis, genetic testing, and the establishment of xenograft models.

**Results:**

Both types of organoids exhibited rapid infiltration when transplanted into the brains of adult immunodeficient mice. However, organoids formed using the microtumor method demonstrated more similar cellular characteristics and tissue structures to the parent tumors. Furthermore, the microtumor method allowed for faster culture times and more convenient operational procedures compared to the Matrigel method.

**Discussion:**

Patient‐derived glioma organoids, especially those generated through the microtumor method, present a promising avenue for personalized treatment strategies. Their capacity to faithfully mimic the cellular and molecular characteristics of gliomas provides a valuable platform for elucidating tumor biology and evaluating therapeutic modalities.

**Conclusion:**

The success rates of the Matrigel and microtumor methods were 45.5% and 60.5%, respectively. The microtumor method had a higher success rate, shorter establishment time, more convenient passage and cryopreservation methods, better simulation of the cellular and histological characteristics of the parent tumor, and a high genetic guarantee.

## Graphical abstract

Organoids can be produced rapidly, frozen, and resuscitated directly. Organoids exhibit rapid infiltration when transplanted in mice brains. Organoids can be rapidly established and used for personalized patient treatment.

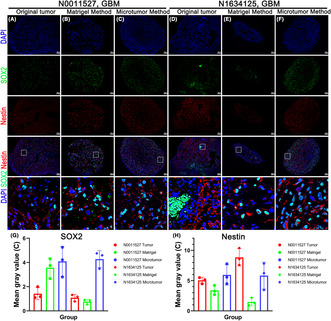

## 1 INTRODUCTION

Glioblastoma (GBM) is the most common malignant primary brain tumor in adults, accounting for 45.6% of all malignant primary brain tumors, with a 5‐year survival rate of 5%.^1^ Although advances have been made in understanding its pathogenesis using the latest sequencing methods, maximal safe surgical resection combined with radiotherapy and temozolomide chemotherapy cannot effectively prolong patient survival. Following treatment, the tumor continues to progress. Patients with GBM have a median survival time of <15 months.^2^

Chemotherapy‐radiation resistance following surgical resection results from GBM cells invading normal brain tissue, making recurrence inevitable owing to the aggressive nature of the cells.^3^ Using single‐cell RNA sequencing, we discovered that individual cells within gliomas express different transcriptional programs associated with oncogenic signals. This suggests intra‐tumoral heterogeneity in GBM. Interpatient heterogeneity leads to distinct outcomes or therapeutic responses in patients at different stages and with different genetic lesions or expression programs.^4^ Consequently, the majority of conventional and targeted therapies fail to be effective in glioma tumors owing to their intra‐ and inter‐tumoral heterogeneity. Hypomethylated MGMT renders temozolomide ineffective.^5^ Furthermore, variable glioma microenvironment can cause tumors to become resistant to therapy and lead to tumor progression. In the microenvironment, tumor cells interact with adjacent stromal cells, including endothelial, stromal, and immune cells, and sustain tumor and an extracellular matrix (ECM), and are influenced by hypoxia, growth factors, cytokines, and nutrients.^6^ Despite research largely focusing on these cells, the complex interactions between tumor cells in distinct subtypes of GBMs and their microenvironment are still in the infancy stage.

For a long time, the development of therapeutics for GBM has largely relied on studies using conventional GBM cell lines, which lack nutrient vessels, oxygen, pH gradients, and the interaction between cells and tumor immune microenvironment (TME). The multiple passage mode of the 2D culture was selected for a population of cells with the ability to proliferate rapidly. Genetic drift significantly affects the similarity between cells—following short‐term culture—and the parent tumor. After a long‐term culture, the cells show progressive genotypic and transcriptomic changes in the parent tumor, and their similarities were transformed.^7^ Tumor stem cells and multicellular tumor spheroids can better retain the metabolic and proliferative gradients of primitive tumors in vivo; however, they are only cell aggregates without complex tissue structures, including the ECM and TME. In addition, they have little histological similarity with the parent tumors and are affected by clonal selection and genetic drift to a certain extent.^8^ To enhance their relevance to human tumors, patient‐derived tumor xenograft (PDX) models transplant tumor samples into mice, which maintain tumor architecture and the relative proportions of cancer and matrix cells. However, PDX models have low transplantation efficiency and success rate. Moreover, they are expensive and may deviate from the primary tumor because of the murine‐specific tumor evolution.^9^ Therefore, a simple and fast model that simulates the organizational structure and properties of the parental tumor tissue is urgently needed to better understand the mechanism of glioma invasiveness in more detail.

Organoids are complex 3D tissue models formed after further in vitro culture of multicellular structures, with the differentiation potential of stem and progenitor cells generated by reprogramming normal or tumor tissues of humans or mice. They can exhibit a structure and function similar to that of the source tissue in a miniature manner. The cerebral organoid model recapitulates the early stages of human brain development. Cerebral organoids exhibit radial glia‐like cells (RDLs) and are used to simulate neurodevelopmental disorders in vitro.^10^ Organoids have also been applied to a variety of tumor models, such as colorectal, lung, pancreatic, breast, liver, ovarian, bladder, and prostate cancers. However, research on tumor organoids in the nervous system remains insufficient. In our study, we established glioma organoid models using two approaches.^11^, ^12^ Furthermore, we analyzed and compared the cellular characteristics, tissue structure, gene expression, and mutation profiles of the organoid models and their parental tumors and summarized the superior culture method.

## 2 MATERIALS AND METHODS

### 2.1 Biological material

#### 2.1.1 Human subjects

Human tissue was used in accordance with the ethical and technical guidelines for the use of human samples and The Code of Ethics of the World Medical Association. Fresh tumor tissue was collected at The First Affiliated Hospital of Soochow University and The Fourth Affiliated Hospital of Soochow University after informed patient consent under a protocol approved by the review board of both hospitals. Cultures were performed within 2 h of tumor resection. All patient samples were diagnosed and graded based on the 2021 WHO Classification of Tumors of the Central Nervous System (CNS), fifth edition.

#### 2.1.2 Experimental animals

Female 6‐week‐old athymic nude mice (BALB/c‐nu nude mouse, Shanghai SLAC Laboratory Animal Co., Ltd, China) were used as experimental animals. The experimental protocol for experimental animals in this study was approved by the review board of The Fourth Affiliated Hospital of Soochow University and complied with the ARRIVE guidelines. The animals were monitored daily for weight loss and physical and neurological abnormalities. The mice were euthanized for brain tissue collection following xenograft establishment.

### 2.2 Medium component

#### 2.2.1 Tumor dissection medium

We combined 490 mL of Hibernate A (Gibco A1247501; Thermo Fisher Scientific, Waltham, MA, USA), 5 mL of GlutaMAX supplement (100×; Gibco 35050061; Thermo Fisher Scientific), and 5 mL of Penicillin‐streptomycin‐amphotericin B Solution (03‐033‐1B; Biological Industries, Beit HaEmek, Israel).

#### 2.2.2 Organoid medium (Matrigel method)

To make the organoid medium, we combined 475 mL Neurobasal medium (Gibco 21103049; Thermo Fisher Scientific), 10 mL B27 minus vitamin A supplement (Gibco 12587010; Thermo Fisher Scientific), 5 mL 200 mM L‐Glutamine (Gibco 25030081; Thermo Fisher Scientific), 5 mL 100 mM Sodium Pyruvate (Gibco 11360070; Thermo Fisher Scientific), 20 ng/mL Epidermal Growth Factor (EGF; Gibco PHG0311; Thermo Fisher Scientific), 20 ng/mL Basic Fibroblast Growth Factor (bFGF; Gibco TL‐401; Thermo Fisher Scientific), and 5 mL Penicillin‐streptomycin‐amphotericin B solution.

#### 2.2.3 Organoid medium (microtumor method)

To make the organoid medium, we combined 235 mL of DMEM/F12 medium (10092026; Corning Inc., Corning, NY, USA), 235 mL of Neurobasal medium, 5 mL of MEM‐NEAAs solution (Gibco 11140050; Thermo Fisher Scientific), 5 mL of GlutaMAX supplement (Gibco 35050061; Thermo Fisher Scientific), 5 mL of Penicillin‐streptomycin‐amphotericin B Solution, 5 mL of N2 supplement (Gibco 17502048; Thermo Fisher Scientific), 10 mL of B27 minus vitamin A supplement, and 125 μL of human recombinant insulin (I9278; Merck, Darmstadt, Germany). Furthermore, we added 10 μM 2‐mercaptoethanol (Gibco 21985023; Thermo Fisher Scientific) to the medium immediately before changing the organoid medium.

#### 2.2.4 Organoid freezing medium

To make the organoid freezing medium, we combined 45 mL of organoid medium (microtumor method), 5 mL DMSO (ST038; Beyotime Biotechnology, Shanghai, China), and 25 μL of 20 mM Y‐27632 dihydrochloride (72304; Cell Signaling Technology, Danvers, MA, USA).

### 2.3 Organoid creation, culture, generation, and biobanking

Please refer to supplementary materials (Data S1) for detailed information.

#### 2.3.1 Matrigel method

Please refer to supplementary materials (Data S1) for detailed information.

#### 2.3.2 Microtumor method

Please refer to supplementary materials (Data S1) for detailed information.

### 2.4 Patient‐derived Orthotopic Xenograft (PDOX) models

Please refer to supplementary materials (Data S1) for detailed information.

### 2.5 Histology, immunohistochemistry, and immunofluorescence

#### 2.5.1 Paraffin embedding and sectioning

Please refer to supplementary materials (Data S1) for detailed information.

#### 2.5.2 Hematoxylin–eosin staining

Please refer to supplementary materials (Data S1) for detailed information.

#### 2.5.3 Immunohistochemical staining

Please refer to supplementary materials (Data S1) for detailed information.

#### 2.5.4 Immunofluorescence staining

Please refer to supplementary materials (Data S1) for detailed information.

### 2.6 Whole exome sequencing (WES) and analysis

Please refer to supplementary materials (Data S1) for detailed information.

## 3 RESULTS

### 3.1 Patient‐derived glioma organoid models were successfully established using microtumor and Matrigel methods

**FIGURE 1:**
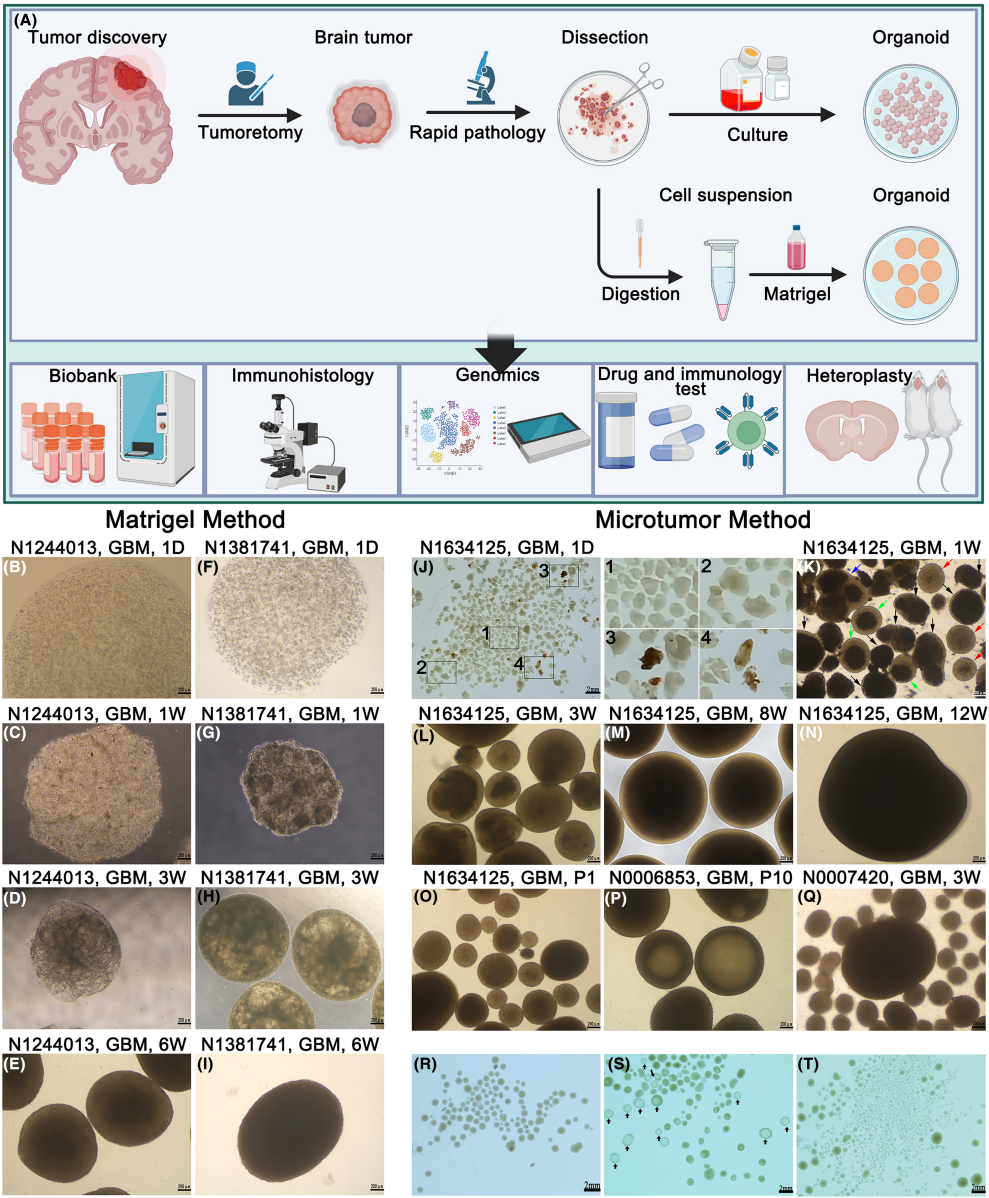
Generation of organoids using Matrigel and microtumor methods. (A) Overview of the organoids generation process from excised tumor tissue and subsequent operations. The panel was created using images from www.biorender.com. (B–E) Light microscope images of the first growth mode of organoids, cultured using the Matrigel method for 1 day, 1 week, 3 weeks, and 6 weeks. Scale bars, 200 μm. (F–I) Light microscope images of the second growth mode of organoids, cultured using the Matrigel method for 1 day, 1 week, 3 weeks, and 6 weeks. Scale bars, 200 μm. (J, R–T) Bright‐field images of organoids, cultured using the microtumor method. Scale bars, 2 mm. (K–O) Light microscope images of the microtumor method for 1 week, 3 weeks, 8 weeks, 12 weeks, and first generation. Scale bars, 200 μm. (P–Q) Two special organoids. Scale bars, 200 μm.

To establish a 3D model that preserves the cell structure and organizational characteristics of the original tumor, we obtained fresh tumor tissue using imaging analysis, neuronavigation, craniotomy, and rapid pathology. Fresh specimens were chopped, digested, diluted with the medium, and mixed with Matrigel to form organoids. Or the specimens were chopped and cultured directly. Figure 1A shows a general flowchart of the organoids from acquisition to subsequent operations. We successfully established 10 and 26 organoids from 23 and 50 specimens using Matrigel and microtumor methods, with success rates of 43% and 52%, respectively. Table 1 present information about the corresponding parental tumors. Successful organoids are characterized by their ability to survive, form dense cell spheroids, and continue to grow. At the initial stage of the study, organoids were mostly cultured using the Matrigel method, which resulted in a huge loss in the cryopreservation and resuscitation processes. Follow‐up samples were cultured by the microtumor method. This results in an uneven distribution between the two methods.

**TABLE 1:** Overview of primary tumor specimen cultivation using microtumor method and Matrigel method.

Sample ID	Age	Sex	WHO grade	Diagnosis	Primary/recurrent	Ki67(%)	IDH mutation	1p/19q co‐deleted	Viable
Microtumor method									
N1591210	69	M	4	Glioblastoma	R	30	NOS	NOS	Y
N1595413	66	M	3	Astrocytoma	P	40	NOS	NOS	Y
N0001689	55	M	4	Glioblastoma	P	1	N	N	N
N0000783	56	M	4	Glioblastoma	P	40	NOS	NOS	N
N0001633	38	M	4	Glioblastoma	P	60	NOS	NOS	N
N0004017	53	M	4	Glioblastoma	P	5	N	N	N
N1615668	48	M	4	Glioblastoma	P	30	NOS	NOS	Y
N0004277	76	F	4	Glioblastoma	P	4	N	N	Y
N1616684	66	F	3	Astrocytoma	P	30	NOS	NOS	Y
N1618394	42	M	4	Astrocytoma	R	15	IDH2 mutation	N	N
N0000378	64	F	4	Glioblastoma	P	20	N	N	N
N0004863	53	F	4	Glioblastoma	P	40	N	N	N
N0284089	45	F	2	Oligodendroglioma	P	2	IDH1 mutation	Y	Y
N0284318	40	F	3	Oligodendroglioma	P	20	IDH1 mutation	Y	Y
N1631521	37	M	3	Astrocytoma	P	10	IDH1 mutation	N	Y
N1632848	77	M	4	Glioblastoma	P	20	N	N	N
N1634125	74	M	4	Glioblastoma	P	60	N	N	Y
N0006853	26	F	4	Glioblastoma	R	20	N	N	Y
N0006894	60	F	4	Glioblastoma	R	50	N	N	Y
N1639293	51	M	4	Glioblastoma	P	40	N	N	N
N0007420	54	F	4	Glioblastoma	P	15	N	N	Y
N1640004	53	F	3	Ependymoma	P	30	N	N	Y
N1643830	71	M	4	Glioblastoma	P	20	N	N	N
N1646757	75	M	4	Astrocytoma	P	10	NOS	NOS	N
N0008848	33	F	4	Glioblastoma	P	40	NOS	NOS	N
N1650548	48	M	4	Glioblastoma	P	40	N	N	N
N0006963	80	M	4	Glioblastoma	P	30	N	N	N
N0488798	80	M	4	Astrocytoma	P	70	NOS	NOS	Y
N0493506	46	M	2	Astrocytoma	P	8	IDH1 mutation	N	Y
N0859318	0.5	M	4	Glioblastoma	P	‐	N	N	Y
N0007929	47	F	4	Glioblastoma	P	20	N	N	N
N0862552	4.5	F	2	Astrocytoma	P	‐	N	N	Y
N0011527	64	F	4	Glioblastoma	P	60	N	N	Y
N0862795	6	F	2	Astrocytoma	P	‐	N	N	Y
N1664993	43	M	3	Astrocytoma	P	30	NOS	NOS	N
N1665945	37	M	4	Glioblastoma	P	80	N	N	N
N0013133	53	M	4	Glioblastoma	P	15	N	N	Y
N0013016	37	M	3	Oligodendroglioma	P	2	IDH1 mutation	Y	Y
N0015822	62	F	4	Glioblastoma	P	40	N	N	Y
N0016140	46	F	4	Astrocytoma	P	30	IDH1 mutation	N	Y
N1006894	61	F	4	Glioblastoma	R	50	N	N	Y
N0021745	58	M	4	Glioblastoma	P	20	N	N	Y
N0028688	71	M	4	Glioblastoma	P	30	NOS	NOS	Y
Matrigel method									
N1381741	64	M	3	Astrocytoma	P	5	NOS	NOS	Y
N1032628	56	M	4	Astrocytoma	P	65	NOS	NOS	Y
N1408446	70	M	3	Astrocytoma	P	15	NOS	NOS	N
N1408491	62	F	3	Astrocytoma	R	5	NOS	NOS	N
N1381288	74	M	4	Astrocytoma	R	40	NOS	NOS	Y
N1416318	62	M	4	Glioblastoma	P	40	NOS	NOS	Y
N1417278	75	F	3	Astrocytoma	P	10	NOS	NOS	N
N1393046	53	M	4	Glioblastoma	P	20	NOS	NOS	Y
N1419042	66	F	3	Astrocytoma	P	5	NOS	NOS	N
N1420249	56	M	3	Astrocytoma	P	15	NOS	NOS	N
N1421933	61	F	3	Astrocytoma	P	5	NOS	NOS	N
N1386912	55	M	4	Astrocytoma	P	30	N	N	N
N1435415	63	M	3	Astrocytoma	P	20	N	N	Y
N1474429	40	F	4	Glioblastoma	R	15	NOS	NOS	N
N1244013	73	M	4	Glioblastoma	P	30	N	N	Y
N1480696	41	M	3	Astrocytoma	P	20	NOS	NOS	N
N1504882	73	M	3	Astrocytoma	P	10	NOS	NOS	N
N1381741	63	M	4	Glioblastoma	P	15	N	N	Y
N1505751	51	M	3	Astrocytoma	P	50	NOS	NOS	N
N1506240	73	M	4	Glioblastoma	P	50	NOS	NOS	N
N1634125	74	M	4	Glioblastoma	P	60	N	N	Y
N0011527	64	F	4	Glioblastoma	P	60	N	N	Y

*Note*: The “Viable” column indicates organoid established successfully at 4 weeks.

Abbreviations: F, females; M, males; N, no; NOS, not otherwise specified; P, primary; R, recurrent; Y, yes.

On the first day of culture using the Matrigel method, tumor cells were independent of each other and evenly distributed in the Matrigel. Next, the cells in the Matrigel began to concentrate slowly. We found that the organoids generated using the Matrigel method showed two growth patterns. First, the tumor cells grew equally in the Matrigel (Figure 1B–D). Second, the tumor cells formed multicenter dense growth areas within the Matrigel. At 1 week, the organoid in Matrigel was concentrated to 1500 μm in diameter. There were several dense areas of cells, approximately 200–300 μm in size, inside the Matrigel, with rough edges. After 3 weeks, the cell‐dense area continued to grow and fuse, and the structure of the whole organoids became dense; however, the size of the organoids did not change significantly (Figure 1F–H). After 4–6 weeks, organoids formed formally with a diameter of approximately 2 mm (Figure 1E,I). Subsequent hematoxylin–eosin (H&E) staining showed that organoids grown according to the first growth pattern (N1244013) had sparser cells than those grown according to the second pattern (N1381741).

On the first day of culture using the microtumor method, we observed ideal tumor fragments, large tumor masses, and pieces with substantial necrosis or hemorrhage (Figure 1J). In the first few days of organoid culture, tissue masses shed a large number of cells, and the medium became cloudy. Organoids were cultivated for 1 week. At this point, it was easy to see which pieces were growing, because they had a round, bright, and dense‐cell form (approximately 500 μm in size) (Figure 1K, red arrow). Some of the visibly black tissue in the central area also showed large proliferation in the surrounding area (Figure 1K, green arrow) and fusion (Figure 1K, blue arrow). We also found that some loose‐like and completely blackened tissue did not grow significantly during culture. These may have been low‐quality tissue masses with complete necrosis (Figure 1K, black arrow). Some proliferating, highly malignant tumors form dense spheroids even on the first day of culture. After the formation of the spheroid structure, the organoids indicated (red arrow in Figure 1K) began to proliferate rapidly, reaching 1–2 mm in size after 3 weeks (Figure 1L). In addition, we found that a few high‐grade and mostly low‐ grade primary tumor fragments did not show significant growth after forming the dense spherical cell structure, and there were no significant changes in the long‐term culture process. Most organoids continued to enlarge, and the central area began to necrose and turn black when visualized under a light microscope (Figure 1M). After 12 weeks, organoids continued to grow to beyond a 2 mm diameter (Figure 1N). We passaged the organoids by cutting them crosswise to form four small pieces at a diameter of approximately 0.5 mm, to reduce core cell apoptosis (Figure 1O,R). The central region of the organoids was restored to bright after passage. This approach preserves the cellular interactions and natural ECM. Interestingly, we found that a cavity‐like structure appeared in the interior of N0006853 after more than 10 generations (Figure 1P,S). We cut up the organoids, which collapsed, and the vacuoles that were filled with culture medium drained out. We also found that N0007420 generated many small organoids without passaging. The small organoids continued to generate after they were removed (Figure 1Q,T).

### 3.2 Both methods retained the histological characteristics of the parents

#### 3.2.1 The cell distribution and anoxic gradient of parent tumors were better simulated by the microtumor method

**FIGURE 2:**
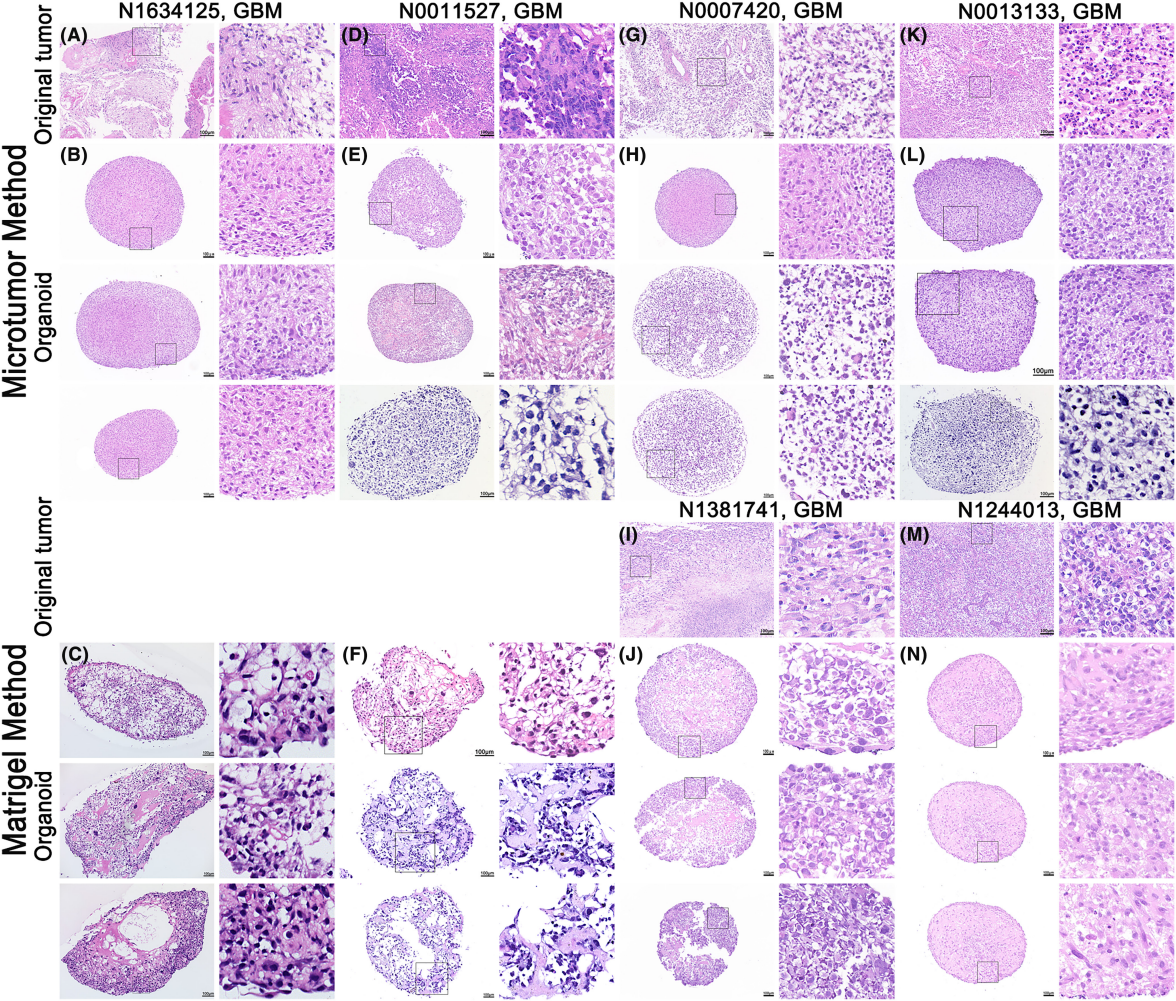
H&E staining of parental tumors and organoids formed using two methods. (A, D, G, K, I, and M) H&E staining results of N1634125, N0011527, N0007420, N0013133, N1381741, and N1244013 parent tumors. Scale bars, 100 μm. (B, E, H, and L) H&E staining results in an organoid at 4 weeks, when cultured using the microtumor method. Scale bars, 100 μm. (C, F, J, and N) H&E staining resulted in an organoid at 4 weeks when cultured using the Matrigel method. Scale bars, 100 μm.

To explore the similarity between the two types of organoids and parent tumors, we first performed histological analyses. We observed nuclear atypia and multinucleation of tumor cells through H&E staining of the original tumor and organoids established using both methods (Figure 2). The cells had a clumped distribution with large nuclei, a high nucleocytoplasmic ratio, and irregular polymorphic nuclei. In addition, the cells were well set outside and the center was sparse. However, according to the H&E staining results, cells cultured using the microtumor method were more closely connected and more densely distributed (Figure 2B,E,H,L). On the contrary, the cells in the organoids cultured using the Matrigel method were arranged more sparsely, and there were large areas of empty cavity structures and barrenness in some of the organoids (Figure 2C,F,J,N).

**FIGURE 3:**
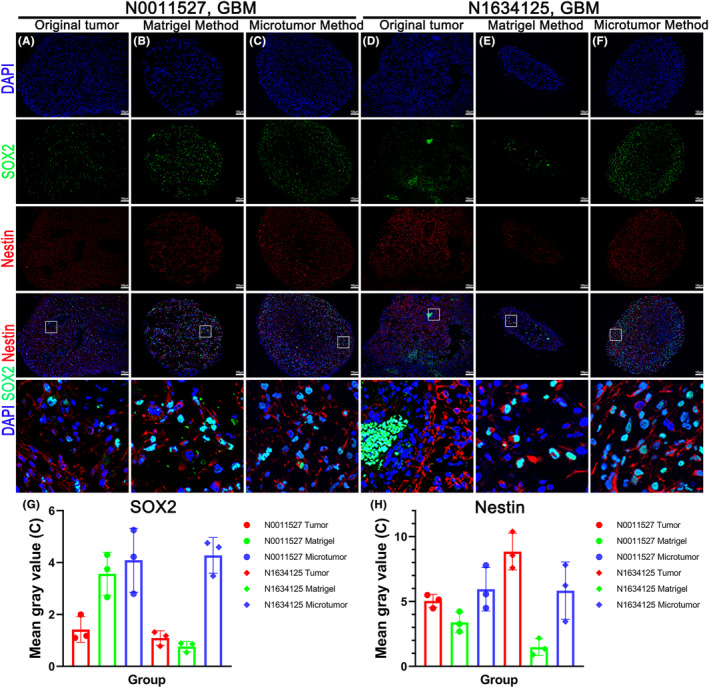
The organoids inherited the stemness markers of the original tumor. (A–F) Immunofluorescence staining images of SOX2 and NESTIN in the original tumor sample and the organoids cultured using the microtumor and Matrigel methods after 4 weeks. Scale bar, 100 μm. (G and H) Quantitative analysis of the immunofluorescence images in (A–F) using ImageJ software.

Organoids are 3D structures composed of multiple cell types that self‐organize from stem cells, and they can mimic the structure and function of native organs. We first investigated whether organoids retain the stem cell characteristics of the parental tumor. SOX genes are developmental regulators that function in the instruction of cell fate and maintenance of progenitor identity during embryogenesis.^13^ Neuroepithelial stem cell protein (NESTIN) is a cytoskeletal intermediate filament originally characterized by neural stem cells. It is now known to be expressed in various tissues and stem or progenitor cells.^14^ SOX2 and NESTIN are widely expressed in both the original tumor and the organoids cultured using the two methods (Figure 3A–F). In sample N0011527, the expression of SOX2 was significantly upregulated in the organoids cultured using both methods, while, in sample N1634125, the expression was slightly lower in the organoids cultured by the Matrigel method but remained enhanced in the microtumor‐cultured samples. As for NESTIN, its expression in sample N0011527 was similar in the organoids cultured using both methods, whereas, in sample N1634125, the organoids cultured by the Matrigel method showed a significant decrease in NESTIN expression. Figure 3G,H shows the corresponding fluorescent quantification analysis.

**FIGURE 4:**
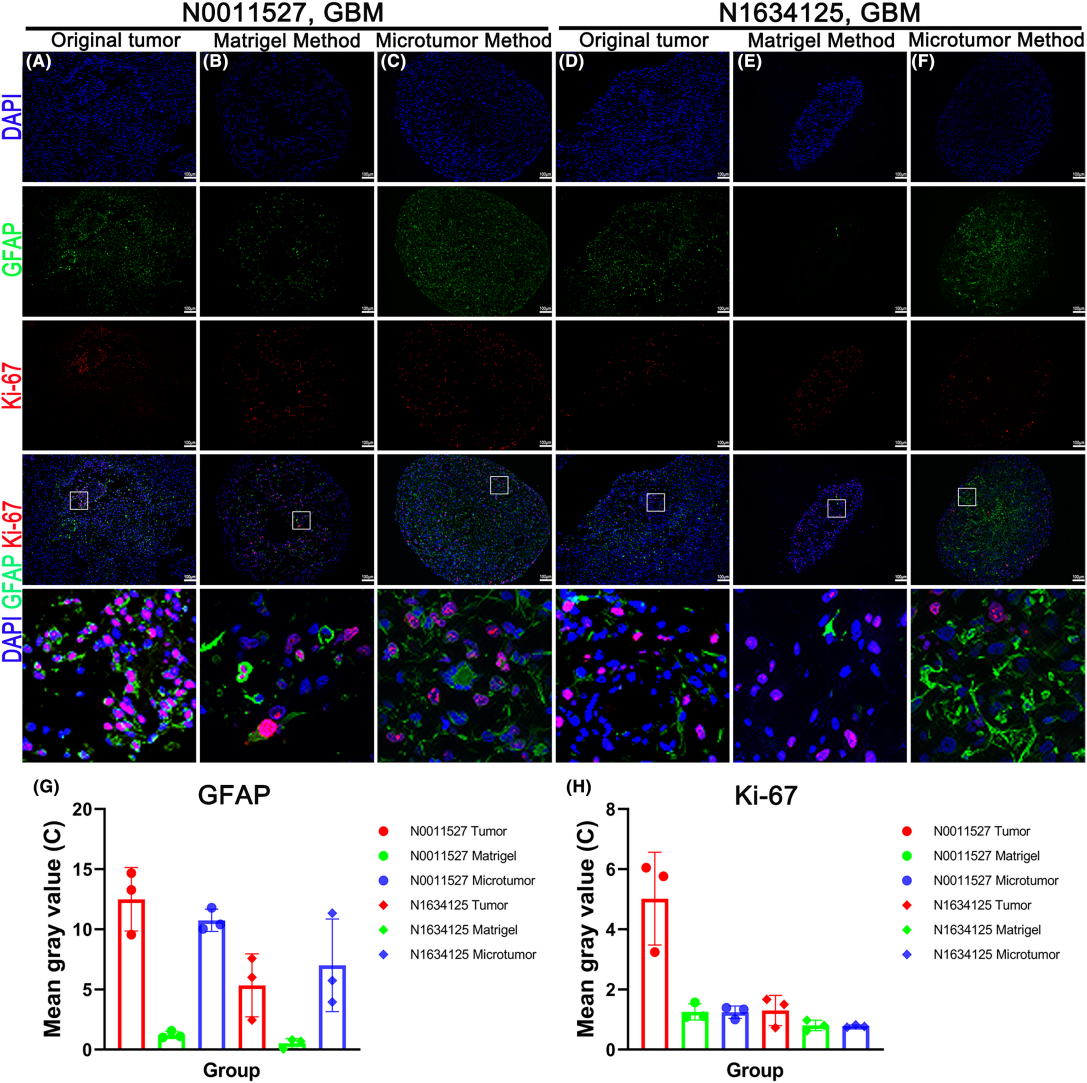
The organoids inherited the glial markers and proliferation indicators from the original tumor. (A–F) Immunofluorescence staining images of GFAP and Ki‐67 in the original tumor samples and organoids cultured using the microtumor and Matrigel methods after 4 weeks. Scale bar, 100 μm. (G and H) Quantitative analysis of the immunofluorescence images in (A–F) using ImageJ software.

To further characterize cell identity in addition to stem cell markers, we performed immunobiological analyses using glial fibrillary acid protein (GFAP). GFAP is a characteristic intermediate filament protein of astrocytes, neural stem cells, and their malignant analogs in gliomas.^15^ We also used Ki‐67 as a proliferative marker to assess cell division within the organoids. Ki‐67 protein expression is related to cell proliferation, differentiation, metastasis, and apoptosis.^16^ Organoids cultured using the microtumor method showed similar GFAP expression to the original tumor, whereas the Matrigel method exhibited a significant decrease. Ki‐67 positive cells in the original tumor exhibited a clustered distribution, while in the organoids, they were widely distributed. The proliferation activity of the organoids cultured using both methods was lower compared to the original tumor (Figure 4A–F). Figure 4G,H shows the corresponding quantitative analysis of the immunofluorescence images.

**FIGURE 5:**
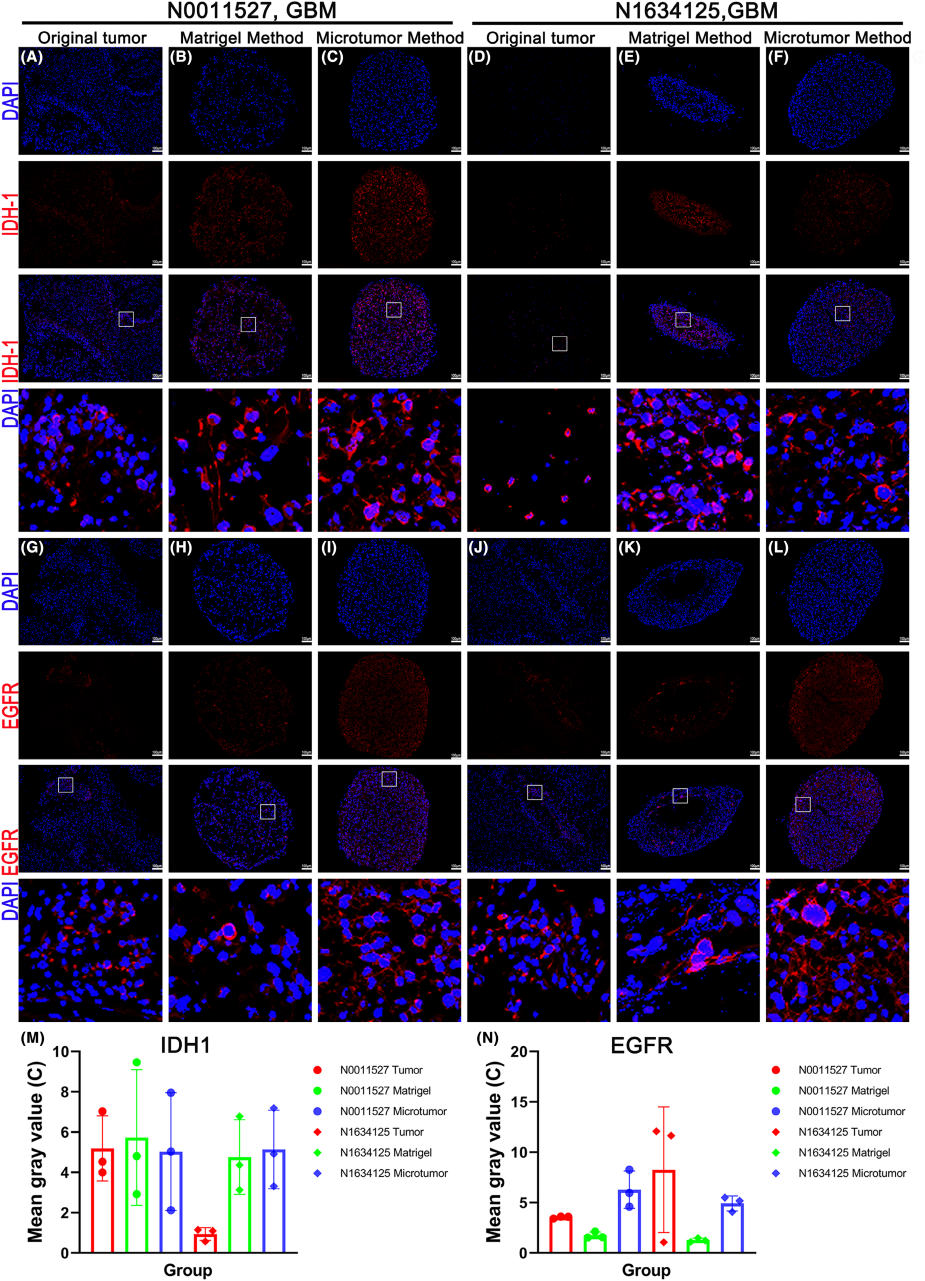
Organoids inherit the characteristic markers of the original tumor. (A–L) Fluorescent immunohistochemistry confocal images of the original tumor sample and organoids cultured using microtumor and Matrigel methods after 4 weeks, stained for IHD1 and EGFR. Scale bars, 100 μm. (M and N) Quantitative analysis of the immunofluorescence images in (A–L) using ImageJ software.

In gliomas, the mutation of IDH1 is closely related to the origin, development, and prognosis of the tumor. The IDH1 gene mutation leads to a change in the function of the enzyme, promoting the accumulation of 2‐hydroxyglutarate (2‐HG) and causing abnormal changes in cellular biological processes such as DNA methylation and cell apoptosis, thereby promoting tumor development and progression. IDH1 mutation is associated with a better prognosis and longer survival.^17^ The epidermal growth factor receptor (EGFR) is a transmembrane receptor tyrosine kinase that is prone to mutations and overexpression. EGFRvIII is a common deletion variant of EGFR, frequently found in GBMs and associated with a worse prognosis. EGFR overexpression refers to a significant increase in the expression level of EGFR in GBM cells, which is associated with tumor invasiveness and recurrence.^18^ We performed immunofluorescence staining for IDH1 and EGFR on the organoid models derived from parental tumors using both the microtumor and Matrigel methods. We observed that the expression of IDH1 was similar in sample N0011527, but in sample N1634125, both methods significantly enhanced the expression of IDH1 (Figure 5A–F). The organoids cultured using the Matrigel method showed a significant decrease in EGFR expression (Figure 5G–L). Figure 5M,N represent the corresponding quantitative analysis of the immunofluorescence images.

**FIGURE 6:**
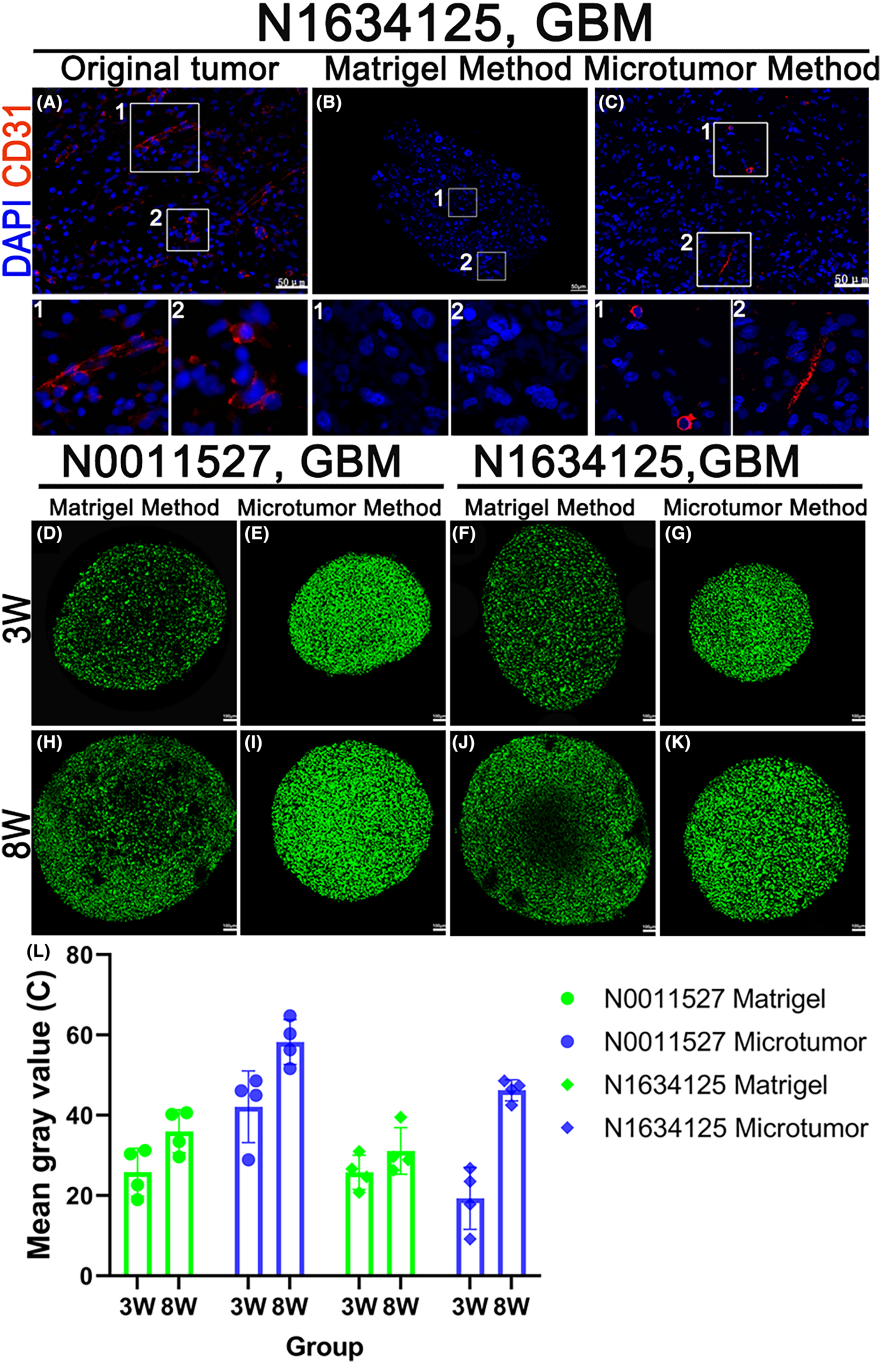
The vascular and proliferation gradients within the organoids. (A–C) Fluorescent immunohistochemistry confocal images of the original tumor sample and organoids cultured using microtumor and Matrigel methods after 4 weeks, stained for CD31. Scale bars, 50 μm. (D–K) Immunofluorescence images of CyQUANT™ Direct Cell Proliferation Assay were used to detect the proliferation within the organoids at 3 and 8 weeks of culture. Scale bar, 100 μm. (M and N) Quantitative analysis of the immunofluorescence images in (D–K) using ImageJ software.

CD31 is a specific marker of endothelial differentiation among non‐hematopoietic human neoplasms and is used to evaluate tumor neovascularization and cancer metastasis.^19^ Venous infiltration is a distinct independent prognostic factor for gliomas. We only found CD31‐positive cells in a small number of organoids cultured using the microtumor method; however, the organoids cultured using the Matrigel method had no obvious CD31‐positive cells (Figure 6A–C). The preservation of vascular structures may be related to the sample selection, and the initial digestion process of the Matrigel method also destroys this structure. We regularly assessed the growth and proliferation of the organoids using the CyQUANT™ Direct Cell Proliferation Assay (C35011; Thermo Fisher Scientific). This assay kit is based on the detection of DNA content and membrane integrity, allowing us to monitor the cell proliferation dynamics within the organoids. Organoids established using the microtumor method showed stronger and denser fluorescence compared to those established using the Matrigel method (Figure 6D–K). In the case of N1634125 organoids cultured using the Matrigel method, the cells in the core region were not stained after 8 weeks (Figure 6J), indicating stagnation in their proliferation.

**FIGURE 7:**
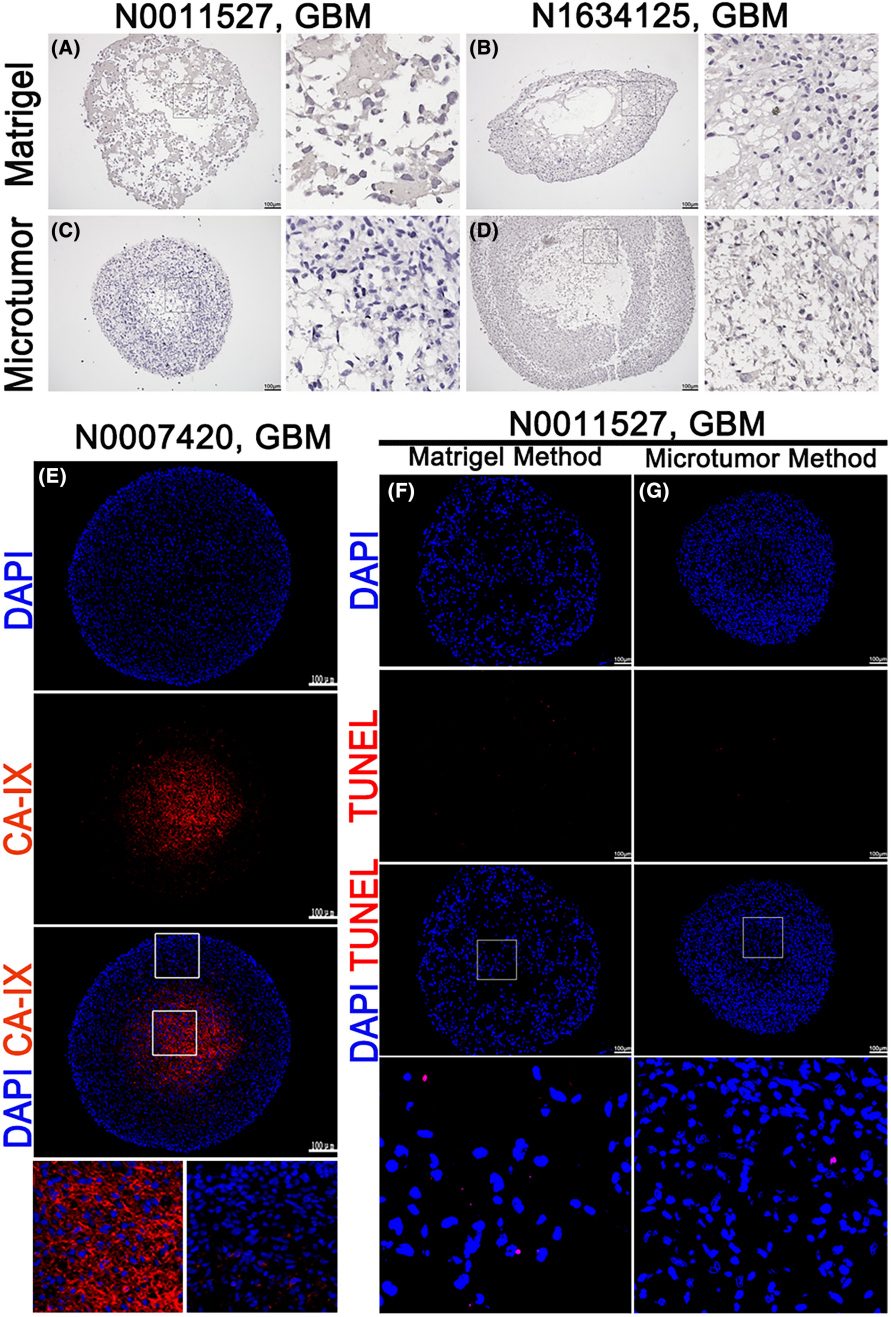
The internal hypoxia and apoptosis within the organoids. (A–D) Immunohistochemical images of the internal hypoxic status within the organoids at 4 weeks of culture using the Hypoxyprobe™‐1 Kit. Scale bar, 100 μm. (E) Fluorescent immunohistochemistry confocal images of organoids cultured using the microtumor method after 4 weeks, stained for CA‐IX. Scale bars, 100 μm. (F and G) Fluorescent immunohistochemistry confocal images of organoids cultured using the microtumor method after 4 weeks, stained for TUNEL. Scale bars, 100 μm.

Hypoxia in glioma stem cells inhibits self‐renewal, proliferation, and survival, and attenuates the tumor initiation potential of glioma stem cells in vivo.^20^ We used the Hypoxyprobe™‐1 Kit (HP1‐100Kit; Hypoxyprobe, Burlington, MA, USA) to assess the hypoxic gradient within the organoids. The specific hypoxia probe (Hypoxyprobe‐1) is minimally expressed or absent in normal cells. However, in hypoxic tumor cells, it is highly expressed, and the intensity of staining deepens with increasing hypoxia severity. We found that N0011527 organoids did not exhibit significant hypoxia (Figure 7A,C). However, both methods of culturing N1634125 organoids resulted in central cavitation, and the microtumor method showed evident hypoxia in the core region (Figure 7B,D). Carbonic anhydrase IX (CA‐IX) is a functional hypoxic marker that is expressed by cells under hypoxic conditions. It is commonly expressed in tumors with the worst prognosis.^21^ We observed that only a very small number of organoids (cultured using the Matrigel method for N0007420) exhibited evident positivity in the core region (Figure 7E), while this expression was not observed in other organoids. Terminal deoxynucleotidyl transferase‐mediated dUTP nick end labeling (TUNEL) assay detects DNA breakage by labeling the free 3′‐hydroxyl termini. In addition, TUNEL staining is widely used to detect apoptosis.^22^ We used the One‐step TUNEL In Situ Apoptosis Kit (E‐CK‐A320; Elabscience, Houston, TX, USA) to detect the apoptosis status within the organoids. Both types of organoids cultured using the two methods showed very few apoptotic cells in their interiors (Figure 7F,G).

#### 3.2.2 The organoid biobank was successfully established by the microtumor method, but challenges persist in the cell recovery by the Matrigel method

Organoids established using the microtumor method can be frozen directly to establish a biobank. Due to the poor permeability of DMSO to the core cells, in advance, organoids were cut into 0.5 mm pieces and pre‐incubated in an organoid freezing medium to enable DMSO to fully infiltrate the core region of organoids. In addition, Y‐27632 (ROCK inhibitor) was used to incubate organoid fragments during cryopreservation and resuscitation, to inhibit cell apoptosis and establish organoid biobanks. After recovery, organoids showed sustainable growth. However, the recovery state of organoids constructed by the Matrigel method was poor after direct cryopreservation, matrix degradation was obvious, and only cells could be isolated and cryopreserved separately after adding the cell detachment solution.

#### 3.2.3 Both methods can quickly establish the PDOX model

**FIGURE 8:**
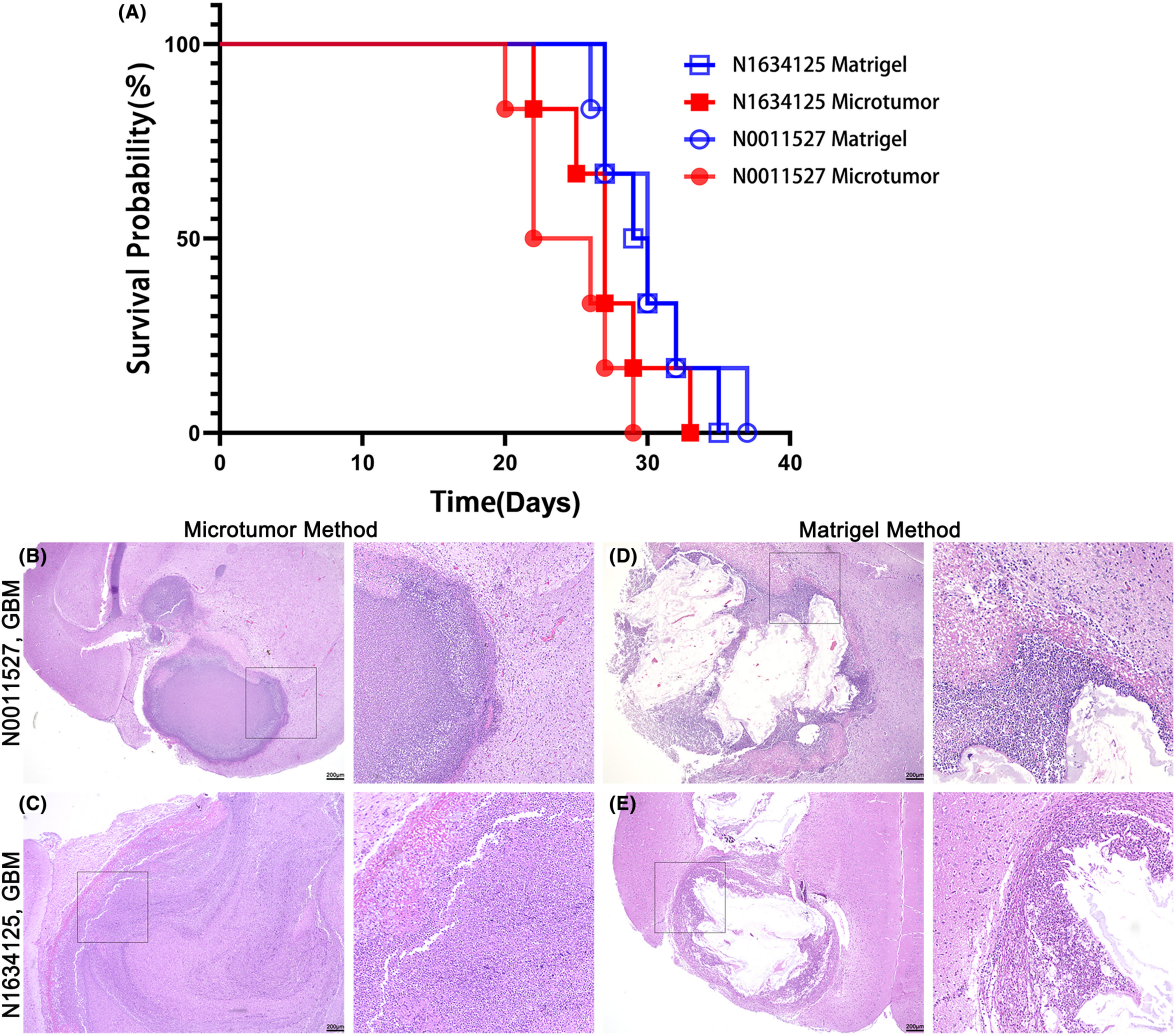
Establishment of PDOX model. (A) Xenograft mouse survival curve: 10 organoids were implanted in each group (*N* = 6, *p* = 0.0183, Gehan–Breslow–Wilcoxon test). (B–E) H&E staining results of the mouse brain. Scale bars, 200 μm.

To investigate the invasiveness of these two methods in xenografts, we transplanted intact organoids into the brains of adult immunodeficient mice at 6 weeks of age.^23^ Each mouse received a transplantation of 10 intact organoids with a diameter of approximately 1000 μm. The PDOX model provides a complete brain microenvironment for tumor transmission, including the structure (vascular system and blood–brain barrier), cells (neurons, glia, microglia, and macrophages), and metabolic components (cerebrospinal fluid and interstitial fluid). Survival curves revealed that the development of brain gliomas in immunodeficient mice took 3–7 weeks (Figure 8A). The average survival period in the PDOX model was 24.3 days for N0011527 organoids cultured using the microtumor method, 30.3 days for N0011527 organoids cultured using the Matrigel method, 27.1 days for N1634125 organoids cultured using the microtumor method, and 30 days for N1634125 organoids cultured using the Matrigel method. Organoids cultured using the microtumor method displayed a shorter survival cycle in the PDOX model. N0011527 organoids cultured using the microtumor method exhibited a shorter survival period compared with N1634125 organoids, possibly influenced by the aggressiveness of the corresponding parental tumor. H&E staining was performed on the brains of sacrificed mice, revealing that the tissue structure at the original transplantation site closely resembled that of the corresponding parental tumors. The transplanted organoids mostly exhibited expansive growth with minimal infiltration (Figure 8B–E). Additionally, we observed that organoids cultured using the Matrigel method were more susceptible to liquefaction necrosis at the center of the graft in the PDOX model (Figure 6E).

#### 3.2.4 The microtumor method completely inherited the genetic mutation of the parent tumor

**FIGURE 9:**
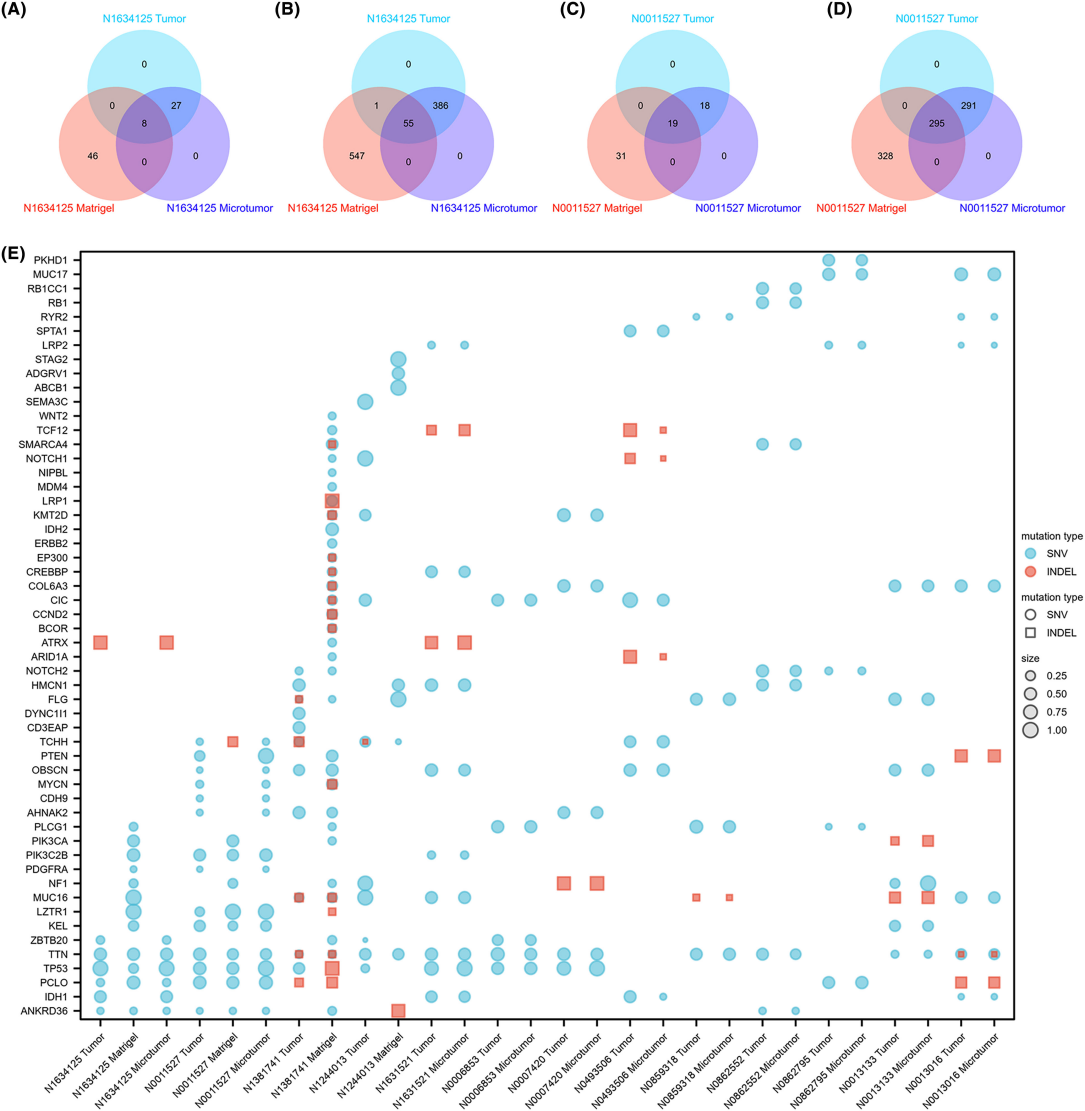
Whole exome sequencing on the organoids and their parents. (A, C) Venn diagrams showing genes with insertion and deletion mutations in the parental tumor and organoids cultured using two methods. (B, D) Venn diagrams showing genes with single nucleotide variations in the parental tumor and organoids cultured using two methods. (E) Variant allele frequency of glioma‐specific gene mutations in organoids and parental tumors.

To further explore whether the organoids retained the genetic characteristics of their parent tumors, we performed whole exome sequencing on the organoids and their parents. The organoids constructed using the microtumor method retained 100% of the single‐nucleotide variants and InDel mutations of the parents. However, the Matrigel method did not maintain 100% consistency with the parent tumor (Figure 9A–D). We screened several genes that are frequently mutated in tumors. The variant allele frequency (VAF) was calculated for each pathogenic single‐nucleotide mutation and insertion–deletion mutation gene. The organoids constructed using the microtumor method and Matrigel method largely retained VAFs similar to those found in the parent tumors (Figure 9E). However, the organoids constructed using the Matrigel method exhibited new mutations in oncogenes.

## 4 DISCUSSION

The infiltration and localization of gliomas in the surrounding normal brain tissue lead to recurrence and death. Therefore, studying the interaction between tumor cells and the ECM is essential for establishing GBM‐specific phenotypes and evaluating therapeutic responses. Traditional tumor models have been unable to satisfy the need for accurate treatment of gliomas. Organoids are 3D structures composed of multiple cell types derived from stem cells through self‐organization, which can simulate the structure and function of native organs. We successfully established a glioma organoid model using the microtumor and Matrigel methods. Cellular heterogeneity within glioma organoids makes it possible to simultaneously culture functionally and phenotypically diverse stem cells with glioma cell populations. Organoids mimic the molecular and cellular properties, tissue structure, gene expression, and mutagenesis of parent tumors in vitro.

We successfully established glioma organoids by direct cutting and resuspension with matrix glue following digestion. In terms of the operational methods, the microtumor method directly creates organoids through mechanical cutting without the digestion steps and retains the most primitive intercellular interactions and intercellular stroma. We observed the spatial distribution of cells inside the organoids, with dense peripheral regions and sparse central regions. Organoids were also established by the Matrigel method using exogenous bFGF, EGF, and artificial matrix. H&E staining showed that the cells were sparsely sliced compared to those of the microtumor method, and there were multiple barren areas inside the organoids. This may be because the original cellular connections were disrupted by the Matrigel method. The establishment time of organoids using the microtumor method was also much shorter than that using the matrix glue method. The artificial matrix cannot completely replace intercellular substances. This microtumor method is convenient for passage and cryopreservation. In addition, it can be cross‐cut into four small pieces to passage directly or cryopreserved after a short incubation period. However, the Matrigel method is very complicated and requires mixing with Matrigel following digestion. The direct freezing state is not good, and only dissociated cells can be frozen.

Next, we discovered through immunohistochemistry that the organoid retained glial cell markers, stem and progenitor cell markers, vascular systems, hypoxia and proliferation gradients, and other GBM‐associated markers from the parental tumor. Stem cell markers were widely expressed in the organoids cultured using both methods. Of note, in sample N0011527, the expression of SOX2 was significantly upregulated in the organoids cultured by both the microtumor and Matrigel methods, indicating successful preservation and enhancement of this marker under the influence of various factors in the in vitro culture. In contrast, for sample N1634125, the expression of SOX2 in the Matrigel‐cultured organoids showed a slight decrease, while it remained enhanced in the microtumor‐cultured organoids. These observations suggested that the microtumor method better preserved the expression of SOX2. NESTIN expression in organoids from sample N0011527 was relatively consistent between both methods of culture. However, in sample N1634125, the Matrigel‐cultured organoids showed a significant decrease in NESTIN expression compared to the original tumor and microtumor‐cultured organoids. The distinct expression patterns of SOX2 and NESTIN in organoids cultured using different methods highlight the potential impact of culture techniques on the preservation of stem cell markers. Furthermore, organoids cultured using the microtumor method displayed GFAP expression similar to that of the original tumor, whereas those cultured using the Matrigel method showed a significant decrease in GFAP expression, indicating that the microtumor method better preserved the glial characteristics of the original tumor. The distribution pattern of Ki‐67‐positive cells within the organoids also differed from that observed in the original tumor. In the original tumor, Ki‐67 positive cells exhibited a clustered distribution, while in the organoids, they were more widely distributed. It should be noted that we deliberately selected regions in the original tumor with a higher distribution of Ki‐67 to present, which resulted in lower fluorescence intensity for both methods of culturing the organoids compared to the original tumor. We found that the expression of IDH1 in sample N0011527 was relatively consistent between the two culturing methods, indicating the successful preservation of this marker. However, in sample N1634125, both methods led to a significant enhancement of IDH1 expression in the organoids. This observation could be attributed to the presence of various non‐tumor components in the original tumor, and during the process of in vitro organoid culture, the proportion of tumor cells might increase, resulting in an upregulation of IDH1 expression. Additionally, the significant decrease in EGFR expression in the organoids cultured using the Matrigel method suggested that the distinct microenvironments and interactions with the ECM in the two culturing systems may influence the expression and signaling pathways of these markers.

In our study, we observed the presence of only a small number of CD31‐positive cells in organoids cultured using the microtumor method, while the organoids cultured using the Matrigel method showed no apparent CD31‐positive cells. The differences in CD31 expression between the two methods can be attributed to several factors. First, the selection of samples for organoid culture may have influenced the preservation of vascular structures. Second, the initial digestion process involved in the Matrigel method may also contribute to the loss of CD31‐positive cells. Pancreatic enzyme digestion could potentially disrupt the delicate vascular network within the tumor, leading to a reduced representation of CD31‐positive endothelial cells in the final organoids. Using the CyQUANT™ Direct Cell Proliferation Assay, we discovered that organoids established using the microtumor method exhibited stronger and denser fluorescence signals compared to those established using the Matrigel method. Additionally, in some Matrigel‐cultured organoids, we observed a lack of staining in the core region after 8 weeks, suggesting that the growth of cells in the core region may be restricted during the process of expansion. This limitation in cell growth could potentially affect the long‐term viability and functional characteristics of Matrigel‐cultured organoids as they continue to grow. In a small subset of organoids established using the microtumor method, we observed clusters of cells positive for both Hypoxyprobe‐1 and CA‐IX in the core region. This suggested that the microtumor method might create a more hypoxic microenvironment within the organoids, resembling characteristics observed in the parental tumor. However, it is important to note that the majority of the organoids did not show significant expression of these hypoxia markers. This indicated that the current culture and passaging protocols supported efficient nutrient and oxygen diffusion within the organoids.

We found that organoids cultured using the microtumor method exhibited a shorter survival period in the PDOX model, which may be related to the cell density and proliferative activity of the organoids. The N0011527 organoids showed a shorter survival period, and this difference could be influenced by the intrinsic invasiveness of the corresponding parental tumor. Histological examination using H&E staining of the brains from sacrificed mice revealed that the tissue structure at the original transplantation site closely resembled that of the corresponding parental tumors. However, the transplanted organoids primarily exhibited expansive growth with minimal infiltration into the surrounding tissue. Additionally, organoids cultured using the Matrigel method were more prone to central liquefaction necrosis in the graft. This observation may be attributed to the poor compatibility between the artificially synthesized Matrigel and the mouse brain tissue, leading to compromised nutrient and oxygen supply in the central region of the organoid graft.

Organoids also showed persistent infiltration in xenograft models, and increasing the number of implants could quickly generate the PDOX model. However, the PDOX model formed using the Matrigel method facilitated the formation of liquefaction necrosis in the infiltrated core area. Whole exome sequencing suggested that organoids cultured using the microtumor method inherited 100% of the genetic mutations in the parent tumors. Both methods retained a similar AVF to that of the parent tumor. This confirms the high genetic fidelity of the organoid model. Organoids may provide a more reliable model for precision medicine by bypassing the barriers to the feasibility posed by in vivo studies.

In our study, the high fidelity of the microtumor method was evident, as the organoids constructed using this approach retained 100% of the single‐nucleotide variants and InDel mutations observed in the parental tumors while maintaining similar VAFs. In contrast, the Matrigel method resulted in the emergence of new oncogene mutations, indicating that the transition from single‐cell suspension to organoids using the Matrigel method may lead to the amplification of certain dominant cell populations and the loss of less‐competitive cell populations, potentially introducing additional genetic alterations not present in the parental tumors. Furthermore, the differences in sequencing regions between the original tumor and the areas used to construct the organoids using the microtumor and Matrigel methods may also contribute to the observed variations in the genetic profile of the tumors. The distinct regions selected for sequencing and organoid construction might harbor different genetic characteristics, influencing the mutation patterns observed in the organoid models compared to the parental tumors.

In conclusion, the success rates of the Matrigel and microtumor methods were 45.5% and 60.5%, respectively (a 15% point difference). The microtumor method had a higher success rate, shorter establishment time, more convenient passage and cryopreservation methods, better simulation of the cellular and histological characteristics of the parent tumor, and a high genetic guarantee (Table 2). The PDOX model had a more similar pattern to the parent tumor.

**TABLE 2:** Comparative analysis of two cultural methods.

Items	Matrigel method	Microtumor method
Source	Patient‐derived tumor specimens	
Treatment	Enzymatic hydrolysis	Cut into pieces
Matrix	Matrigel	Natural extracellular matrix
Pattern of cultivation	Suspension culture of cell‐Matrigel mixture	Suspension culture of tumor pieces
Organoid composition	Tumor cells and Matrigel	Tumor cells, endothelial cells, immune cells and stromal cells
Condition of culture	37°C, 5%CO_2_, 90%RH, 120 rpm shaker	
The same composition of the medium	Neurobasal, B27, glutamine, antibiotic	
Different compositions of medium	EGF, bFGF, sodium pyruvate	DMEM: F12, MEM‐NEAAs, N2, insulin
Cycle of growth	3–6 weeks	1–3 weeks
Size	1–2 mm	0.5–2 mm
Passage	Digestion and blending with Matrigel	Cross‐cutting
Cell density	Sparse	Dense
Cryopreservation condition	Freeze‐digested single cells	Cross‐cut and incubate briefly
Proliferation	+	+
Stem cell properties	+	+
Glial cell marker	+	+
Hypoxia gradient	+	−
Nutrient vessel	+	−
Apoptosis	Few	Few
PDOX model	Simulate the parent tumor	Liquefaction necrosis in the center
WES	100%	Emergent mutation
VAF	Similar	Similar
Success rate	45.5% (10/22)	60.5% (26/43)

Abbreviations: VAF, variant allele frequency; WES, Whole Exome Sequencing.

However, our organoid model also has some limitations. First, although our success rate has improved compared to that at the initial stage of the study, an ideal result is yet to be achieved. Second, in the process of culture, we found that the quality of organoids may depend on the degree of malignancy and the quality of the tumor tissue obtained. Third, organoids are more likely to be formed from the parent tumor margin without burning. Therefore, close cooperation between surgeons and pathologists is key to obtaining high‐quality organoids. Therefore, we are exploring further studies.

## AUTHOR CONTRIBUTIONS

**Yang Zhang:** Conceptualization (equal); data curation (lead); formal analysis (equal); funding acquisition (equal); investigation (equal); methodology (equal); project administration (equal); visualization (lead); writing – original draft (lead). **Yunxiang Shao:** Conceptualization (equal); data curation (equal); investigation (equal); methodology (equal); software (equal); validation (equal). **Yanyan Li:** Formal analysis (supporting); investigation (supporting). **Xuetao Li:** Data curation (supporting); investigation (supporting); supervision (supporting); validation (supporting). **Xuewen Zhang:** Data curation (supporting). **Qinzhi E.:** Resources (supporting); validation (supporting). **Weichao Wang:** Resources (supporting); validation (supporting). **Zuoyu Jiang:** Resources (supporting); validation (supporting). **Yulun Huang:** Funding acquisition (lead); project administration (lead); resources (lead); software (equal); supervision (equal); writing – review and editing (lead). **Wenjuan Gan:** Funding acquisition (equal); project administration (equal); supervision (equal).

## FUNDING INFORMATION

This work was supported by grants from the National Natural Science Foundation of China (grant numbers 82173279, 82102770); the National Science and Technology Resources Shared Services Platform Project (grant number YCZYPT [2020]06‐1); General Research Project of Jiangsu Provincial Health Commission (grant numbers H2017064, H201621: M2022050); and Applied Research on Key Technologies for the Well‐being of Suzhou Citizens (grant number SS201864).

## CONFLICT OF INTEREST STATEMENT

The authors declare there is no conflict of interest in the study.

## Supporting information


Data S1.

## Data Availability

The data that support the findings of this study are available on request from the corresponding author. The data are not publicly available due to privacy or ethical restrictions.
