# Supplementary material for: The generation of glioma organoids and the comparison of two culture methods
Source: Cancer Med. 2024 Mar 8;13(4):e7081. doi: 10.1002/cam4.7081 (PMC10923046; doi:10.1002/cam4.7081)
Supplement: Supplementary file 1 — Data S1. [file CAM4-13-e7081-s001.docx]

**MATERIALS AND METHODS**

**Organoid Creation, Culture, Generation, and Biobanking**

The following were the common first steps for organoid creation:

1. After the diagnosis of glioma was verified by radiology and rapid pathology, the tissue was placed in 5 mL of tumor dissection medium kept on ice and brought to the laboratory for treatment within 2 h.
2. The tumor dissection medium was removed from above the tumor and the tumor tissue was washed three times with 10 mL of precooled PBS, and the supernatant was removed.
3. An appropriate amount of tissue was transferred to a 6-well plate. Using sterilized ophthalmic scissors (Shi Qiang Group Limited, jyj1030), the tissue was cut into larger tissue chunks and distributed into the wells of the 6-well plate. The tissue chunks were immersed in 100 μL of tumor dissection culture medium. The plate was tilted at a 45° angle, allowing the tissue chunks to settle at the bottom of the wells. Using ophthalmic scissors, the tissue chunks were cut into fragments with a diameter of approximately 0.5–1 mm.
4. The tumor tissue was washed three times in 10 mL of PBS at room temperature. The tumor chunks were allowed to settle at the bottom of a conical tube, then the supernatant was carefully removed.
5. 10 mL of red blood cell lysis buffer (C3702; Beyotime Biotechnology) was added and the mixture was gently shaken on a benchtop shaker at room temperature for 10 min.
6. The red blood cell lysis buffer was removed and the samples were washed with 10 mL of pre-warmed DMEM/F12 medium three times.

***Matrigel Method***

For preparation with Matrigel, the following additional steps were followed:

1. The paraffin sealing film (1337416; Thermo Fisher Scientific) was cut in advance to the desired size and placed between two PCR reaction plates, gently pressing to create consistent wells. This was disinfected with 70% ethanol and placed in the culture dish. This was then exposed to UV light overnight. Frozen Matrigel (Corning, 354234) was thawed at 4 °C and 100-μL pipette tips were placed in a pre-chilled -20 °C freezer.
2. The tumor fragments were transferred to a new culture dish. Trypsin (Gibco 25200056; Thermo Fisher Scientific) was added at volume 4–5 times that of the tumor sample. Digestion proceeded at 37 °C in a sterile incubator for 30–45 min with gentle tapping every 10 min.
3. When the tissue fragments in the culture dish visibly disappeared, the cells were sequentially filtered using sterile 70-μm and 40-μm filters. After centrifuging at 1000 rpm for 5 min, the cells were resuspended in the organoid medium.
4. A counting plate was used to count the total number of cells in the cell suspension. The cell suspension was mixed with four times its volume of Matrigel on ice. Using a pre-chilled pipette tip, 20 µL of the cell suspension were added to the wells created by pressing the paraffin sealing film between the two PCR reaction plates. Each well contained approximately 20,000 cells.
5. After incubating the 20 µL of cell suspension at 37 °C for 1–2 h until it solidified, it was transferred into a 6-well plate. 4 mL of organoid medium were added to each well, and samples were cultured in a sterile incubator at 37 °C, 5% CO_2_, and 90% relative humidity.
6. After 4 d, the 6-well culture plate was transferred to an orbital shaker rotating at 120 rpm.
7. The following steps were performed for passaging and biobanking:

(a) The organoids were transferred to a 15-mL conical tube and the supernatant was aspirated. The organoids were washed once with a tumor dissection culture medium.

(b) Approximately 300 μL of trypsin were added per 10 organoids, and samples were incubated in a 37 °C incubator for 5–10 min, with gentle tapping every 3 min.

(c) When the organoid fragments had visibly diminished, an equal amount of organoid medium was added, following by centrifuging at 1000 rpm for 5 min. Steps 10–12 were then repeated to establish the organoids again.

(d) Alternatively, the above cell suspension was resuspended in 1 mL of organoid freezing medium, transferred into cryovials, and labeled with the relevant information. Finally, the cryovials were placed in an isopropanol freezing container and a controlled freezing process was initiated at -8037 °C. The next day, the cryovials were transferred to liquid nitrogen for long-term storage.

1. For recovery:

(a) The cryovials were removed from the liquid nitrogen and quickly thawed in a 37 °C water bath, gently rotating the tubes to ensure even thawing. When only a small piece of ice remained, vials were removed from the water bath. The external surface of the vials was disinfected with 70% ethanol.

(b) The cell suspension was transferred to a 15-ml conical tube. The suspension was Slowly diluted with an organoid culture medium containing DMSO, centrifuged at 1000 rpm for 5 min, and then resuspended in organoid culture medium. The organoids were then re-established following steps 10–12.

***Microtumor Method***

For forming organoids using the microtumor method, the following steps were followed.

1. The tumor pieces were placed in a 6-well culture plate in 4 mL of organoid medium, and the plate was placed in an orbital shaker rotating at 120 rpm at 37 ℃, 5% CO_2_, and a 90% humidity sterile incubator. The culture plate was tilted at 45° to replace 75% of the medium every 1 to 4 d, according to the medium color changes. In the long-term culture process, we found that the medium color changes could be used as a reference for medium replacement. The medium color of neonatal organoids tends to change from red to yellow quickly. Delayed medium replacement may result in cell shedding and even organoid lysis. This suggests that we can only cultivate a few organoids per well.
2. Passaging: When organoids reach a diameter of 1–2 mm, it is necessary to passage them to prevent extensive necrosis in the core cells. For this:

(a) the organoids were transferred to a 15-mL conical tube, the supernatant was aspirated, and they were washed once with tumor dissection medium.

(b) The organoids were transferred to a 6-well culture plate and immersed in 100 μL of tumor dissection medium. Using conjunctival scissors (y20000; Shi Qiang Group Limited, Zhejiang, China), the organoids were cross-cut into small pieces (approximately 0.5 mm in diameter), dividing them into four parts.

(c) The organoid fragments were collected in a 15-mL conical tube and washed three times with 10 mL of room temperature DMEM/F12 medium.

(d) The organoid fragments were placed in a 6-well culture plate with 4 mL of organoid medium and incubated on an orbital shaker rotating at 120 rpm in a sterile incubator at 37 ℃, 5% CO_2_, and 90% relative humidity.

1. Biobanking:

(a)–(c) Same as above

(d) The organoid fragments were placed in a 6-well culture plate with 4 mL of organoid medium supplemented with 10 μM Y-27632. The plate was incubated on an orbital shaker rotating at 120 rpm in a sterile incubator at 37 ℃, 5% CO_2_, and 90% relative humidity for 1 h.

(e) The organoid fragments were collected in a 15-mL conical tube and resuspended in 10 mL of organoid freezing medium. The tube was incubated on a rocking platform shaker at room temperature for 10 min.

(f) After discarding the supernatant, the organoid fragments were resuspended in 2 mL of organoid freezing medium.

(g) Twenty organoid fragments were transferred to each cryovial, adding enough freezing medium to reach a total volume of 1 mL; the vials were labelled with the relevant information.

(h) Finally, the cryovials were placed in an isopropanol freezing container and a controlled freezing process was initiated at -80 ℃. The next day, the cryovials were transferred to liquid nitrogen for long-term storage.

1. Recovery:

(a) The cryovial was rapidly thawed in a 37 °C water bath, gently rotating the tube to ensure uniform thawing. Once only a small piece of ice remained, the cryovial was removed from the 37 °C water bath and the external surface was disinfected it with 70% ethanol.

(b) T/he organoid fragments were transferred to a 15-ml conical tube and the DMSO was slowly diluted with 10 ml of organoid medium containing 10 μM Y-27632.

(c) The supernatant was then collected and 2 ml of organoid medium containing 10 μM Y-27632 were added.

(d) The organoid fragments were placed in a 6-well culture plate and 4 ml of organoid medium containing 10 μM Y-27632 were added, followed by incubation overnight in a sterile incubator at 37 °C, 5% CO_2_, and 90% humidity.

(e) The next day, the medium was replaced with regular culture medium and incubation was continued on an orbital shaker rotating at 120 rpm in a sterile incubator (88881102; Thermo Fisher Scientific) at 37 °C, 5% CO_2_, and 90% humidity.

**Patient-derived Orthotopic Xenograft (PDOX) Models**

1. We ground the tips of the spinal needles and sterilized them at high temperature in advance. Under a microscope, organoids that were approximately 1000 μm in diameter were collected, and they were transferred to a 15-ml conical tube. they were then washed three times with 10 ml of room temperature DMEM/F12 medium and placed on ice.
2. Mice were anesthetized using isoflurane gas and each mouse's head was fixed in a stereotaxic frame (#68001; RWD Life Science, Shenzhen, China). Anesthesia was maintained using isoflurane delivered through a face mask.
3. Using long forceps, the organoids were pick up one by one and placed into the head end of the lumbar puncture needle. The needle was secured in the syringe holder of the stereotaxic frame.
4. The skin on the mouse's head was disinfected with 75% ethanol and a longitudinal incision approximately 0.7 cm in length was made slightly posterior to the bregma. Using a 2.0-mm electric grinder bit (DZ070; QINIU, Shenzhen, China), a small hole was created approximately 2 mm posterior to the right side of the mouse's fontanelle.
5. Using the stereotaxic instrument, the tip of the spinal needle was positioned in the small hole, ensuring it was in close contact with the surface of the mouse's brain. The needle was then slightly lowered by 2.5 mm, and left in place for 2 min before being slightly retracted by 0.5 mm and slowly injecting the organoids. The needle was kept in place for 2 min and then slowly removed. Using bone wax (#AR9763; Johnson & Johnson, New Brunswick, NJ, USA), the cranial window was closed and the skin was sutured.

**Histology, Immunohistochemistry, and Immunofluorescence**

***Paraffin Embedding and Sectioning***

1. Tissue Collection: The organoids were fixed in 4% paraformaldehyde for over 24 h and placed in a dehydration box.
2. Dehydration and Paraffin Infiltration: Dehydration was performed in an alcohol gradient (75% for 4 h, 85% for 2 h, 90% for 2 h, 95% for 1 h, 100% I for 30 min, 100% II for 30 min) using a tissue processor. Then, the organoids were incubated in xylene (I for 10 min, II for 10 min at 65 °C) and then in melted paraffin wax (I for 1 h, II for 1 hat 65 °C, III for 1 h).
3. Embedding: First, a small amount of liquid paraffin was added to the embedding cassette. Before it solidified, the tissue was placed in the cassette and it was filled with liquid paraffin, followed by solidification at -20 °C.
4. Sectioning: The paraffin block was fixed onto a paraffin microtome and 4- μm sections were cut. The sections were placed on a tissue flotation bath at 40 °C to flatten the samples. The flattened tissue samples were transferred onto glass slides, dried in a 60 °C oven, and then stored at room temperature.

***Hematoxylin & Eosin Staining***

1. Deparaffinization and Hydration: The slides were baked on a hot plate for 1 h and then sequentially placed in xylene (I for 20 min, II for 20 min) and ethanol (100% I for 5 min, 100% II for 5 min, 75% for 5 min).
2. Hematoxylin Staining: The sections were soaked in hematoxylin staining solution for 3–5 min, followed by rinsing with water. The sections were submerged in the differentiation solution for 1 min and rinsed with water again. Finally, the sections were dipped in the bluing reagent for 1 min and rinse with water.
3. Eosin Staining: Sections were sequentially placed in ethanol (85% for 5 min, 95% for 5 min) after dehydration and then immersed in the eosin staining solution for 5 min.
4. Dehydration: The sections were sequentially placed in ethanol (100% I for 5 min, 100% II for 5 min) and xylene (I for 5 min, II for 5 min).
5. Mounting: Apply a neutral mounting medium to the slides.

***Immunohistochemical Staining***

1. Deparaffinization and Hydration: After baking the slides on a hot plate for 1 h, they were sequentially placed in xylene (I for 15 min, II for 15 min, III for 15 min) and ethanol (100% I for 5 min, 100% II for 5 min, 85% for 5 min, 75% for 5 min). The slides were then rinsed once with distilled water.
2. Antigen Retrieval: The sections were placed in a glass container filled with sodium citrate antigen retrieval buffer, ensuring that they were submerged. The buffer was boiled in a high-temperature sterilizer at 100 °C for 30 min and naturally cooled for 30 min. The sections were place on a rocking shaker and washed with PBS 3 times for 5 min each time.
3. Blocking Endogenous Peroxidase: The tissue sections were circled with a hydrophobic pen. The sections were incubated with 3% hydrogen peroxide solution for 25 min in darkness. Then, they were washed on a rocking shaker with PBS 3 times for 5 min each time.
4. Blocking Endogenous Antigens: Blocking was performed using 3% bovine serum albumin at room temperature for 1 h.
5. Primary Antibody Incubation: The blocking solution was discarded, and the pre-prepared primary antibody solution was added. Slides were incubated overnight in a humidified chamber at 4 °C.
6. Secondary Antibody Incubation: The primary antibody solution was discarded, and the slides were washed on a rocking shaker with PBS 3 times for 5 min each. After washing, the corresponding secondary antibody solution was added, and samples were incubated at room temperature for 50 min in a humidified chamber.
7. DAB Staining: The secondary antibody solution was discarded, and slides were washed on a rocking shaker with PBS 3 times for 5 min each. After washing, DAB staining solution was added, and samples were incubated for 8 min. The slides were then rinsed with running water.
8. Hematoxylin Counterstaining: The sections were soaked in hematoxylin staining solution for 3–5 min and then rinsed with water. The sections were submerged in the differentiation solution for 1 min and rinsed with water again. Finally, the sections were immersed in the bluing reagent for 1 min and rinsed with water.
9. Dehydration: The sections were sequentially dehydrated in ethanol (75% for 5 min, 85% for 5 min, 100% I for 5 min, 100% II for 5 min) and xylene for 5 min.
10. Mounting: The sections were air-dried and mounted with coverslips and neutral mounting medium.

***Immunofluorescence Staining***

1. Deparaffinization, Hydration, Antigen Retrieval, Blocking Endogenous Antigens, Primary Antibody Incubation, and Secondary Antibody Incubation: These steps are described above in steps 1–6 in *Immunohistochemical Staining*.
2. DAPI Counterstaining: The sections were washed on a rocking shaker with PBS 3 times for 5 min each. After washing, excess liquid was removed, and DAPI staining solution was added to the sections. Sections were incubated at room temperature for 10 min in a light-protected environment.
3. Mounting: The sections were washed on a rocking shaker with PBS 3 times for 5 min each. After washing, excess liquid was removed and the sections were mounted with a coverslip using an appropriate mounting medium (G1401; Servivebio, Wuhan, China).
4. Images were acquired using a fluorescence microscope (BZ-X810; Nikon, Tokyo, Japan) and assembled manually using the software NIS_F_Ver43000_64bit_E (Nikon) and Adobe Photoshop 2022 (Adobe, San Jose, CA, USA).

**Whole Exome Sequencing and Analysis**

1. Nucleic Acid Extraction: DNA was extracted from the original tumor and organoids using a HiPure Universal DNA kit (Magen Biotechnology, Guangzhou, China).
2. Library Sequencing: Qualified DNA samples were collected and a focused ultrasonicator (S220; Covaris, Woburn, MA, USA) was used to randomly fragment 200 ng of gDNA into fragments smaller than 300 bp. Subsequently, end repair was performed (including 5' end phosphorylation and 3' end adenylation) and sequencing adapters were ligated. Fragments of approximately 300 bp in size (with the inserted amplification fragment size around 200–250 bp) were selected using magnetic bead purification. Finally, library amplification was performed using P5 and P7 primers. The amplified products were purified using magnetic beads to obtain the library. Probes, hybridization buffer, and blocker were added to the 750 ng library, and hybridization was performed for less than 24 h. The hybridization products were then washed with wash buffer to remove non-specific binding products, followed by library amplification enrichment and library quality control. The DNA libraries, labeled with different indexes, were mixed and subjected to PE150 paired-end sequencing according to the instructions of the HiSeq Xten/Novaseq/MGI2000 instrument (Illumina, San Diego, CA, USA). After data generation, the data were analyzed.
3. Data Analysis: After obtaining the raw data with sufficient sequencing depth, bioinformatics analysis was performed.
4. Sequencing Data Quality Analysis: Next, the raw data were preprocessed using Cutadapt software (v1.9.1) to remove primer and adapter sequences, trim sequences with base quality below 20 at both ends, and filter out sequences with an N base ratio greater than 10%. The cleaned data that passed quality control were then subjected to statistical analysis.
5. Data Alignment: Cleaned data were aligned, sorted, and deduplicated against a reference genome using Sentieon (v202010-02) or BWA (v0.7.12) to generate BAM files.
6. Variant Detection: Single nucleotide variants/InDel variants were detected using Sentieon or GATK HaplotypeCaller (v3.5), and the variants were annotated using Annovar (v2016/5/11).
7. Copy Number Variation Detection: Copy number variation detection was performed using Control-FREEC (version v10.6).
